# Occurrence of Fetal Macrosomia Rate and Its Maternal and Neonatal Complications: A 5-Year Cohort Study

**DOI:** 10.5402/2012/353791

**Published:** 2012-11-14

**Authors:** Mahin Najafian, Maria Cheraghi

**Affiliations:** ^1^Department of Obstetrics & Gynecology, School of Medicine, Ahwaz Jundishapur University of Medical Sciences, Ahwaz 61357-15794, Iran; ^2^Department of Public Health, Ahwaz Jundishapur University of Medical Sciences, Ahwaz 61357-15794, Iran

## Abstract

*Background*. Macrosomia is defined as an infant's birth weight of more than 4000 g at term which is to different maternal and neonatal complications. Several studies have been done on factors influencing risk of macrosomia, but there is lack of information and study in our country regarding macrosomia complications. *Objective*. The aim of this study was to determine the prevalence of macrosomia and its complications. *Method*. A cohort study was conducted from 2007 to 2011 at Obstetrics and Gynecology Department, Razi Hospital in Ahvaz city, Iran. All pregnant mothers who were referred to Obstetrics and Gynecology Department for delivery were included in this study. The total number of 201,102 pregnant mothers was recruited and divided into case and control groups after delivery (macrosomia (case) and normal weight infants (control) groups). *Results*. Out of total deliveries (201,102), there were 1800 macrosomia, (9%). Gestational diabetes, maternal obesity (BMI), maternal aged and positive history of previous macrosomia were the major risk factors for macrosomia which were compared with the normal weight infant groups (*P* < 0.001 for all parameters). Neonatal complications associated with macrosomia included humerus—clavicle fractures and arm—brachial plexus injury which were significant compared to the control group (*P* < 0.001 for all parameters). *Conclusion*. The macrosomia is potentially dangerous for the mother and the neonate. It is important to recognize the suspected fetal macrosomia to prevent its risk factors and complications. There is a need to provide all delivery facilities and care services to prevent and reduce the maternal and neonatal macrosomia complications.

## 1. Introduction

Macrosomia is described as a newborn with an excessive birth weight. Fetal macrosomia has been defined in several different ways, including birth weight of 4000–4500 g (8 lb, 13 oz to 9 lb, 15 oz) or greater than 90% for gestational age after correcting for neonatal sex and ethnicity (90th percentile) [1]. A diagnosis of fetal macrosomia can be made only by measuring birth weight after delivery; therefore, the condition is confirmed only after delivery of the neonate. Fetal macrosomia is encountered in up to 10% of deliveries [1].

Morbidity and mortality associated with macrosomia can be divided into maternal, fetal, and neonatal categories. A study investigating the effects of birth weight on fetal mortality shows that higher fetal mortality rates are associated with a birth weight of greater than 4250 g in nondiabetic mothers and a birth weight of 4000 g in diabetic mothers [2]. Factors associated with fetal macrosomia include genetics, duration of gestation, presence of gestational diabetes, and diabetes mellitus types I and II. Genetic, racial, and ethnic factors influence birth weight and the risk of macrosomia [3]. Maternal diabetes is one of the strongest risk factors associated with giving birth to an infant that is considered large for gestational age. Pregestational and gestational diabetes result in fetal macrosomia in as many as 50% of pregnancies complicated by gestational diabetes and in 40% of those complicated by type 1 diabetes mellitus. Studies of macrosomic infants of diabetic mothers revealed a greater amount of total body fat, thicker upper-extremity skin fold measurements, and smaller ratios of head to abdominal circumference than macrosomic infants of nondiabetic mothers [4].

Maternal weight prior to pregnancy or obesity can affect the weight of the fetus. Women who are obese are more likely to have larger infants [5]. Excessive weight gain in pregnancy is a risk factor for macrosomia. The risk is greater for women with obesity than for women without obesity [5]. Multi-parity and grand multi-parity increase the risk of macrosomia [6]. Parity has been reported to be associated with 100–150 grams of weight gain at birth [7]. 

Macrosomia neonates are at risk for shoulder dystocia and birth trauma. This risk is directly related to neonatal birth weight and begins to increase substantially when birth weight exceeds 4500 g and particularly when it exceeds 5000 g. Brachial plexus injury is rare, with an incidence of fewer than 2 cases per 1000 vaginal deliveries. This risk is approximately 20 times higher when the birth weight is more than 4500 g [8]. Stillbirth rates in macrosomic infants are twice as high as those in control subjects, irrespective of diabetes. However, for a birth weight of 4500–5000 g, the fetal death rate is fewer than 2 deaths per 1000 births for nondiabetic women and is approximately 8 deaths per 1000 births for diabetic women. For a birth weight of 5000–5500 g, this rate is 5–18 deaths per 1000 births for nondiabetic women and is approximately 40 deaths per 1000 births for diabetic women [2]. 

Race and ethnicity are associated with macrosomia. Macrosomia occurs with higher frequency in newborns of Hispanic origin. Because Hispanic women have a higher incidence of diabetes during pregnancy, part of the preponderance of macrosomia in this ethnic group is due to the higher incidence of diabetes in pregnancy. However, even when corrected for diabetes, Hispanic mothers tend to have larger newborns.

Fetal sex influences macrosomia potential. Male infants weigh more than female infants at any gestational age. Recent studies have confirmed this association [9]. Excessive amniotic fluid defined as greater than or equal to 60th percentile for gestational age has recently been associated with macrosomia [10]. Despite all these risk factors for macrosomia, much of the variation in birth weights remains unexplained. Most infants who weigh more than 4500 g have no identifiable risk factors.

## 2. Methodology

### 2.1. Study Design and Location

A cohort study was conducted at Obstetrics and Gynecology Department, Razi Medical and Educational Center, Ahvaz, Iran, during the years 2007–2011. The study was approved by the Jundishapur University of Medical Sciences and Ethics Committee.

### 2.2. Subjects

There were about 20,000 live births during 5 years. They were divided into two groups (i.e., macrosomia (case) and normal birth weight (control) groups). The inclusion criteria were those with singleton pregnancy regardless to maternal age, number of parity, type of previous delivery, and agreement to participate. The exclusion criteria were incomplete subjects' profiles. The subjects were matched with respect to personal characteristics. We obtained the written informed consent form from the research subject.

### 2.3. Measurements

The outcomes measurements were maternal characteristics and medical history (i.e., gestational diabetes, pregnancy-induced hypertension), delivery details (e.g., birth order, number of live children, number of stillbirths, number of intrauterine deaths, postterm delivery, type of previous delivery (normal vaginal delivery, Cesarean section)), nutritional status (i.e., BMI), and maternal and perinatal morbidity data. 

### 2.4. Statistical Analysis

Data was analyzed using SPSS, version 16. The entire test was 2 sided significant at the level of 0.05 by estimating power of 95%. Normality test was performed, and all variables were normally distributed. The chi-square (*χ* ^2^) test with considering odds ratio (OR) was performed. Fisher's exact test was performed for variables with frequency less than 5 in each cell of 2 × 2 table. 

## 3. Results

Out of 20,000 live-birth deliveries, 1800 (9%) infants had birth weight of about 4000 g and above. A number of 1520 (7.6%) infants had birth weight between 4000 to 4499 gr and 1.2% (*n* = 240) had birth weights between 4500 to 4999 g. Only 0.2% had birth weight about 5000 gr or more (Table 1).

Sixty percent (60%) of mothers and having macrosomic infants-aged 35 years and above. About fifty-nine (59.5%) of subjects and having macrosomic infants were of Arab ethnicity. There was significant association between macrosomia and diabetes (*P* < 0.05). The results showed that 712 (39.5%) of diabetes subjects delivered macrosomia neonates. Also there was significant association between maternal obesity and macrosomic infants (*P* < 0.05). Maternal obesity was a risk factor for macrosomia. Out of 1637 obese pregnant mothers, 1350 (75%) delivered macrosomic neonates. a significant association between multiparity and macrosomia (*P* < 0.05) was observed. About 81% (*n* = 1458) of macrosomic neonates were delivered from multiparity mothers. There was a significant association between positive history of previous macrosomia and postterm delivery (*P* < 0.05 for all parameters) (Table 2). The results also revealed that out of 675 Cesarean sections, 89% (*n* = 162) delivered macrosomic neonates (Table 2). This indicates that macrosomia increases the risk of Cesarean section.

The maternal complications due to macrosomia were uterine atony (*n* = 211), cervix and vaginal laceration (*n* = 92), and uterine rapture (*n* = 10). There were significant associations between macrosomia and uterine atony, cervix/vaginal laceration and uterine rapture (*P* < 0.05 for all parameters) (Table 3). The results showed that neonatal complications included brachial plexus injury (*n* = 37), and a clavicle fracture (*n* = 14). There was a significant association between macrosomia and brachial plexus injury and clavicle fracture (*P* < 0.05 for all parameters). About 180 macrosomic infants had first-minute Apgar score of less than 6 which was significant compared to normal birth weight infants (*P* < 0.05). Brachial fracture was observed in 6 macrosomic infants that were significant with normal birth weight neonates (*P* < 0.05). Shoulder dystocia was observed among 192 neonates that 11% (*n* = 183) were macrosomia fetus (Table 3).

## 4. Discussion

The study successfully evaluated the prevalence rate of macrosomia and its maternal and neonatal complications during 5 years. This study showed that the incidence of macrosomia was 9% during 5 years. The result was comparable with the study conducted by Ehrenberg et al. in 2004 in Ohio, US, that found that the prevalence of macrosomia was 11.8% of population sample [11]. The incidence of macrosomia also was confirmed to be approximately 7%–10% [12]. The newborns that were 4500 g or heavier constituted 1%-2% of all of the newborns [12]. The incidence of macrosomia was reported as 9.8% in a study conducted in Turkey [13]. However, the rate was determined as 5.8% in the study by Hajy-Ebrahim-Tehrani et al. in Iran [14]. The ratio of the newborns of 4500 g and heavier was 0.9% [14].

The current study showed that maternal age of 35 years and older was 60% in macrosomia group compared to control group (21%) and is a risk factor for macrosomia. It was comparable with 9.6% in a study by Hajy-Ebrahim-Tehrani et al. [14]. Maternal diabetes rate among macrosomia and control groups was 39.5% versus 6.1%, respectively. In another study, the incidence of gestational diabetes was about 1%–3% in the population [15]. It was recorded 1% [16]. The incidence of gestational diabetes is reported 1%-2% in the mothers of macrosomic babies. This incidence is about 5%–7% with births of 4500 g and heavier [12, 17]. 

Our study pointed out that maternal obesity was associated with macrosomia, and it was 75% in the study group as compared to the control group (16%). Obese women were more at risk for macrosomic infants' delivery. It was noted in another study that obese mothers were at elevated risk for large for gestational age (LGA) delivery as compared to normal weight mothers (16.8% versus 10.5%,  *P* < 0.0001) [11]. Multiparity was significantly associated with macrosomia group compared to control group (81% versus 34%, *P* < 0.05) which was comparable with (13.2% versus 9.5%, *P* < 0.0001) [11].

In the current study the result revealed that history of a previous macrosomia in macrosomic group was 18% compared to control group (1.3%). There are many studies reporting that the history of a pervious macrosomic baby is the most common leading maternal factor to macrosomia [17]. The result that also supported by Hajy-Ebrahim-Tehrani et al. study that showed the history of previous macrosomic baby was ten times higher in the macrosomic birth group [14].

The incidence of Cesarean section in the macrosomic group of our study was 89% compared to the control group (11%). The Caesarean delivery is justified in all cases of fetal weight estimation greater than 4500 g [18]. The rate of Caesarean section significantly increased among the patients who delivered after labour induction as compared to those whose labour was not induced [19]. The rate of cesarean section among women delivering macrosomic babies was 47.6% in Saudi Arabia [20].

It was observed in the current study that maternal complications such as uterine atony (11%), cervix/vaginal laceration (4.9%), and uterine rapture (0.4%) were significantly associated with macrosomia. Another study involved in Saudi Arabia found that the most common maternal complications were postpartum hemorrhage (1.2%), perineal tear (1.7%), and cervical lacerations (0.7%) [20]. The risk of postpartum bleeding and genital tract injury was about 3–5 times higher in macrosomic deliveries in another study [21]. The risk of genital laceration and atony was observed to be significantly higher [14]. 

The current study pointed out that prevalence of shoulder dystocia, brachial plexus injury, clavicle fracture, and brachial fracture among macrosomic neonates was 11%, 1.9%, 0.6%, and 0.3%, respectively. Our result was confirmed by 9.6% for shoulder dystocia, 0.96% for Erb's palsy and 1.4% for bone fracture [20]. It was considerable that the incidence of birth trauma was increased 3 times in the macrosomic group. The newborns with birth weight 4500 g or heavier carried six times higher risk of birth trauma [14]. In a study completed in Parkland Hospital, the rate of brachial paralysis was 4.737 in deliveries between 4000–4500 g and 4.118 in the deliveries of 4500 g and over in a total of 1162 macrosomic birth [22].

## 5. Conclusion

Although no intervention has been proven to significantly reduce the risk of macrosomia, several potentially useful strategies may be helpful. In diabetic patients, tight glucose control before pregnancy can reduce the risk of congenital malformation. In both diabetic mothers and in those with gestational diabetes, tight control during pregnancy with the use of diet and insulin can reduce the frequency of macrosomia. Prevention of maternal obesity before pregnancy may reduce the frequency of macrosomia. The rate of perinatal and maternal morbidity can be reduced by the antenatal diagnosis. The risk factors leading to macrosomia must be thoroughly evaluated by the clinician. Since the majority of factors which lead to the delivery of macrosomic infants are preventable, it is hoped that with close cooperation of gynecologists, pediatricians and dieticians along with training of mothers, the number of such incidences would be minimized.

## Figures and Tables

**Table 1:** Frequency of macrosomia according to birth weight (data presented as number (*n*) and percentage (%)), (*n* = 1800).

Birth weight (g)	Number (*n*)	Percentage (%)
4000–4499	1520	7.6
4500–4999	240	1.2
≥5000	40	0.2

**Table 2:** Comparison of maternal risk factors between case and control groups (data presented as number (*n*) and percentage (%)).

Variables	Macrosomia (case)		Normal birth weight (control)		
	*n*	%	*n*	%	*P*
Diabetes (*n* = 820)	712	39.5	108	6.1	0.0001
Obesity (BMI ≥ 25 kg/m^2^) (*n* = 1637)	1350	75	287	16	0.0001
Multiparity (*n* = 2070)	1458	81	612	34	0.005
History of macrosomia (*n* = 347)	324	18	23	1.3	0.0001
Postterm delivery (*n* = 241)	180	10	61	3.4	0.005
Maternal big frame size (*n* = 1086)	774	43	312	18	0.001
Maternal age (≥35 years) (*n* = 1457)	1080	60	377	21	0.005
Family history (*n* = 340)	306	17	34	1.9	0.0001
Ethnicity (Arab) (*n* = 2027)	1071	59.6	956	53	0.72
Cesarean section (*n* = 675)	162	89	513	28.5	0.32
Normal vaginal delivery (*n* = 1485)	198	11	1287	71.5	0.26

*P* < 0.05 is significant using chi-square test.

**Table 3:** Maternal and neonatal complications due to macrosomia (data presented as number (*n*) and percentage (%)).

Variables	Macrosomia		Normal birth weight		
	*n*	%	*n*	%	*P*
Uterine atony (*n* = 211)	198	11	13	0.7	0.0001
Cervix/vaginal laceration (*n* = 92)	88	4.9	4	0.2	0.0001*
Uterine rapture (*n* = 10)	8	0.4	2	0.1	0.0001*
Shoulder dystocia (*n* = 192)	183	11	9	0.5	0.0001
Brachial fracture (*n* = 6)	6	0.3	0	0.0	0.0001*
Clavicle fracture (*n* = 14)	12	0.6	2	0.1	0.0001*
Brachial plexus injury (*n* = 37)	35	1.9	2	0.1	0.0001*
First-minute Apgar < 6 (*n* = 193)	180	10	13	0.7	0.0001

*P* < 0.05 is significant using chi-square test, *P < 0.05 is significant using Fisher's exact test.
